# *Medusa*: A Novel Gene Drive System for Confined Suppression of Insect Populations

**DOI:** 10.1371/journal.pone.0102694

**Published:** 2014-07-23

**Authors:** John M. Marshall, Bruce A. Hay

**Affiliations:** 1 Medical Research Council Centre for Outbreak Analysis and Modelling, Department of Infectious Disease Epidemiology, Imperial College London, London, United Kingdom; 2 Division of Biology and Biological Engineering, California Institute of Technology, Pasadena, California, United States of America; Instituto de Higiene e Medicina Tropical, Portugal

## Abstract

Gene drive systems provide novel opportunities for insect population suppression by driving genes that confer a fitness cost into pest or disease vector populations; however regulatory issues arise when genes are capable of spreading across international borders. Gene drive systems displaying threshold properties provide a solution since they can be confined to local populations and eliminated through dilution with wild-types. We propose a novel, threshold-dependent gene drive system, *Medusa*, capable of inducing a local and reversible population crash. *Medusa* consists of four components - two on the X chromosome, and two on the Y chromosome. A maternally-expressed, X-linked toxin and a zygotically-expressed, Y-linked antidote results in suppression of the female population and selection for the presence of the transgene-bearing Y because only male offspring of *Medusa*-bearing females are protected from the effects of the toxin. At the same time, the combination of a zygotically-expressed, Y-linked toxin and a zygotically-expressed, X-linked antidote selects for the transgene-bearing X in the presence of the transgene-bearing Y. Together these chromosomes create a balanced lethal system that spreads while selecting against females when present above a certain threshold frequency. Simple population dynamic models show that an all-male release of *Medusa* males, carried out over six generations, is expected to induce a population crash within 12 generations for modest release sizes on the order of the wild population size. Re-invasion of non-transgenic insects into a suppressed population can result in a population rebound; however this can be prevented through regular releases of modest numbers of *Medusa* males. Finally, we outline how *Medusa* could be engineered with currently available molecular tools.

## Introduction

Population suppression is an important tool for controlling the unwanted effects of insect pests and disease vectors, many of which cause extensive damage to agricultural crops and transmit infectious diseases to plants, animals and humans such as malaria and dengue fever [1], [2]. A variety of tools are available to suppress insect populations, such as insecticides, hormone-baited traps and the introduction of sterile males; however, all of these techniques are expensive and require a substantial, ongoing effort in order to maintain suppression [1]. For malaria, the availability and distribution of long-lasting insecticide-treated bed nets and artemisinin-based combination therapy drugs have led to reductions in transmission in many countries [2]; however it is not expected that these tools will be sufficient to eliminate the disease from highly-endemic areas [3]. The problem is exacerbated by the emergence of drug resistant forms of *Plasmodium* [4], and insecticide resistance among vector species [5]. For dengue fever, there are currently no drugs or vaccines available and so control measures are limited to suppression of the vector species *Aedes aegypti* [6]. Consequently, there is continuing interest in the development of novel genetics-based strategies for insect population management [7]–[11]. One strategy is to utilize gene drive systems to spread genes into wild mosquito populations that make their bearers unable to transmit diseases [9]–[11]. Here, we focus on an alternative strategy whereby genetic approaches are used to suppress insect populations.

Genetic population suppression strategies fall into two broad categories – those that are self-limiting and inundative, and those that are self-propagating. In self-limiting strategies, sterile males, or males carrying transgenes that reduce the number of female progeny, are released to mate with wild females, thus reducing the female population and hence the total population size [8], [11]–[13]. These strategies are self-limiting because the sterile insects/transgenes are only expected to persist for a few generations following their release; however they also require frequent and large-scale releases over an extended period, making them challenging for large-scale control programs such as malaria control in Africa. That said; releases of radiation-sterilized insects have successfully eliminated agricultural pests such as the Mediterranean fruit fly and New World screwworm on huge scales [12] and field trials of genetically sterile males have successfully demonstrated suppression of *Ae. aegypti* populations in the Cayman Islands [14], [15], indicating there are contexts in which these strategies can succeed.

In self-propagating strategies, a gene drive mechanism is used to spread transgenes into a population at the same time as it suppresses the population. Two mechanisms have been proposed to bring this about. In one approach, known as Y-drive, genes located on the Y chromosome are used to create a bias towards viable spermatozoa carrying the Y chromosome rather than the X. The resulting gamete segregation distortion produces a male-biased gender ratio, eventually causing the population to crash due to a lack of females [16], [17]. Systems displaying these properties, generated through linkage of a naturally-occurring segregation distortion system with the *Drosophila* Y chromosome, have been shown to drive population extinction in laboratory populations of *Drosophila* [18]. Recent efforts have been directed at creating synthetic Y-drive through expression of site-specific nucleases, known as X-shredders, which cleave the X chromosome of Anopheline mosquitoes at multiple sites during spermatogenesis, also leading to Y-biased sperm production [19].

In a second approach, homing endonucleases (HEGs) are engineered that recognize a target site on versions of the homologous chromosome lacking the HEG. If homologous recombination copies the HEG to the cut chromosome, this leads to an increase in HEG frequency in subsequent generations [11]. If the target site is located within a gene required in somatic tissues for female fertility or viability, then spread of the HEG may result in a decrease in mosquito density and disease transmission [20], [21]. HEG-based gene drive has recently been demonstrated in the malaria vector *Anopheles gambiae* in a proof-of-principal, engineered genetic background [22].

Self-propagating population suppression systems face competing mandates. On the one hand, they must be capable of spreading genes that reduce population fitness to high frequency, while on the other hand, their spread must be restricted according to social and regulatory contexts that are currently being defined [23]–[25]. Central to these discussions are the issues of safety, confinement and reversibility. Y-drive and HEGs are predicted to be highly invasive [11], [20], [26], which is appealing for wide-scale control as transgenic insects migrating from one population to another can potentially cause a cascade of population crashes. However, it would be challenging to prevent the spread of these transgenes across international borders [24] or to guarantee the confinement of isolated field trials [27]. A strategy has been proposed to reverse the spread of a deleterious HEG through the release of HEG-resistant alleles in the event of unforeseen consequences [11]; however resistant alleles may have to target multiple waves of population crashes while not entirely restoring the pre-transgenic state.

Given the scrutiny that transgenic insects have faced to date [25], we argue there is a need for gene drive systems capable of bringing about a reversible population crash in a confined region. Drive mechanisms with these characteristics would enable the ecological effects of a self-propagating population suppression system to be tested prior to a release on a wide scale. Additionally, there may be cases where population suppression restricted to a local environment is the goal. In these cases, a gene drive system that induces population suppression locally, but that is unable to spread to high frequency in surrounding regions, would have much smaller release and maintenance requirements than a sterile male approach.

**Figure 1:**
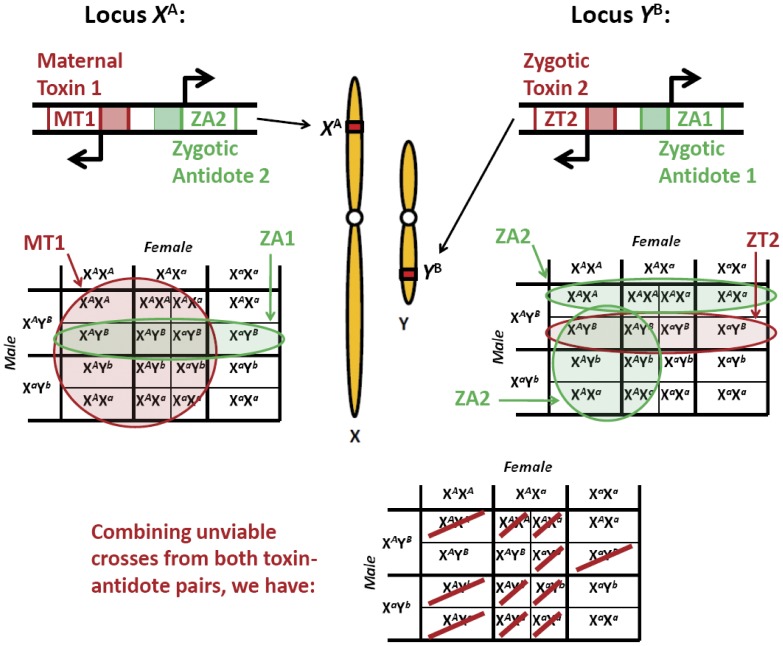
Components and inheritance pattern of the *Medusa* system. *Medusa* consists of four components – two at a locus on the X chromosome and two at a locus on the Y chromosome. The action of the maternally-expressed, X-linked toxin is suppressed in zygotes that inherit the Y-linked antidote. The effects of the zygotically-expressed, Y-linked toxin are suppressed in zygotes inheriting the X-linked antidote.

Here, we propose a novel gene drive system, *Medusa*, which displays these properties. The system consists of four components - two at a locus on the X chromosome and two at a locus on the Y chromosome (Fig. 1). The combination of a maternally-expressed, X-linked toxin and a zygotically-expressed, Y-linked antidote causes suppression of the female population and selects for the transgene-bearing Y since only transgenic male offspring of *Medusa*-bearing females are protected from the effects of the toxin. At the same time, the combination of a zygotically-expressed, Y-linked toxin and a zygotically-expressed, X-linked antidote selects for the transgene-bearing X when the transgene-bearing Y is present. Together these chromosomes create a balanced lethal system that, when present above a threshold frequency, spreads while creating a strong male gender bias. A detailed description of how insects with these transgenes are generated and maintained is provided later in the manuscript. The name *Medusa* is an anagram for “sex chromosome-associated *Medea* underdominance”, as its components are identical to those of *Medea*, which consists of a maternal toxin and zygotic antidote [28], and engineered underdominance, which consists of two alternate pairs of zygotic toxins and antidotes [29]. The name also has origins in Greek mythology, where *Medusa* is a beautiful yet terrifying woman who causes onlookers to be turned to stone (toxin); but was ultimately beheaded by Perseus who distracted himself with Athena’s mirrored shield (antidote).

The ability to confine *Medusa* to a local environment stems from its threshold dynamics. That is, for deterministic models, a threshold frequency exists above which the system spreads into a population and induces a crash, and below which it is eliminated from the population. For stochastic models, an effective threshold exists above which the system is very likely to spread, and another exists below which it is very likely to be eliminated (these approach the deterministic threshold at high population sizes). Systems displaying this property are confineable to partially-isolated populations provided migration rates are sufficiently low that they never exceed the effective threshold frequency in neighboring populations [30]. Here, we present simple population genetic models that describe the dynamics of the *Medusa* system in randomly-mating populations, with special reference to the malaria vector, *An. gambiae*. We explore the potential utility of *Medusa* as a tool for confined population suppression, with comparisons to alternative strategies, and discuss molecular tools that could be used to engineer *Medusa* in the laboratory.

## Results

The modelling framework described in the Methods can be used to explore the dynamic properties of the *Medusa* system and its utility for confined suppression of mosquito populations. We begin by using a discrete-generation, deterministic population frequency framework to explore the system’s threshold properties – i.e. the frequency at which *Medusa* must be present in order to spread into a population. We then use a discrete-time, stochastic framework to model the possibility of a population crash following a super-threshold release. This framework is modified from that used by Deredec *et al.* [21] to describe the dynamics of HEGs and incorporates the egg, larval, pupal and adult life stages, with overlapping generations and density-dependent mortality at the larval stage, since larval competition is reduced at low population sizes, allowing a single female to produce more offspring that survive to adulthood. We then extend this latter model to a metapopulation system of two partially-isolated populations, into one of which *Medusa* males are introduced. This allows us to explore the hypothesis of confined population suppression – i.e. suppression in the release population while the neighboring population is left unchanged. Finally, we use this framework to compare *Medusa* to female-specific RIDL [31] and autosomal X-shredders [20] – two other genetic population suppression systems that provide an intermediate between sterile male releases and invasive population suppression systems. For female-specific RIDL, the lethal gene is not expressed in males and hence the lethal gene and resulting population suppression is sustained over several generations [31]. For autosomal X-shredders, the population is biased towards males and the autosomal gene and hence male gender bias is sustained over several generations; however there is no gene drive as for Y-linked X-shredders [20]. Of note, female-specific RIDL is well-developed as a tool for population suppression in *Ae. aegypti* [31]; while autosomal X-shredders are currently being developed for *An. gambiae*.

### Single population dynamics

The key to confined population suppression with *Medusa* is the existence of a deterministic threshold frequency, above which the system spreads and reduces the number of female offspring, and below which it is eliminated. All-male releases are preferred for mosquitoes since males don’t bloodfeed and so are unable to spread vector-borne diseases. An all-male release also has the benefit that it doesn’t increase the female population size. A single release of X*^A^*Y*^B^* males (*A* represents the transgenic allele on the X chromosome, *B* represents the transgenic allele on the Y chromosome, and *a* and *b* represent the corresponding wild-type alleles) is insufficient for gene drive since, as Fig. 1 reveals, the only viable transgenic offspring are X*^A^*X*^a^* females, and these produce no viable offspring upon mating with wild-type males. However, if a second release of X*^A^*Y*^B^* males is carried out, viable transgenic offspring are produced and the system is capable of spreading into the population.

**Figure 2:**
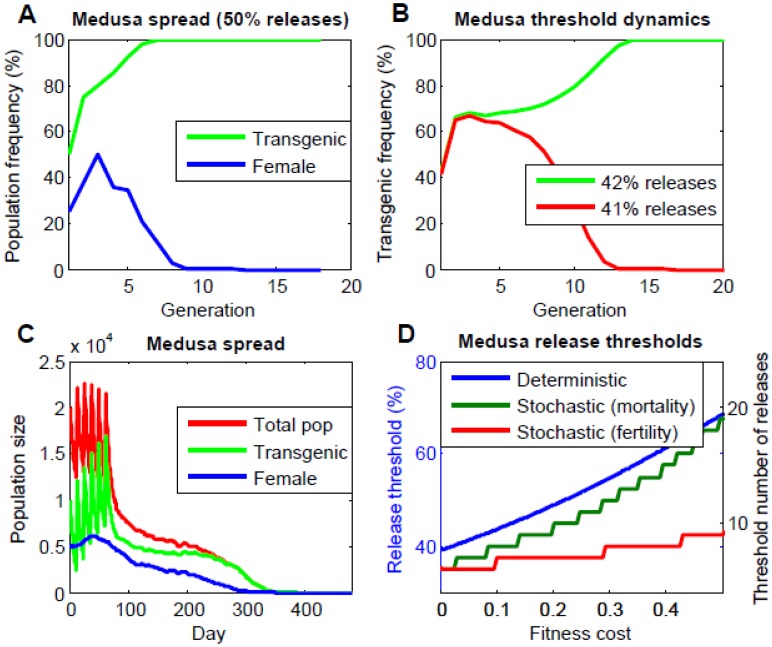
*Medusa* dynamics in a single population. A: Gene drive and elimination of females following two consecutive releases of males carrying the *Medusa* system (no fitness cost) at a population frequency of 50% (i.e. released males represent 50% of the population post-release for each of the two releases). B: Threshold properties of the *Medusa* system (no fitness cost) – two consecutive releases of males at a population frequency of 42% results in gene drive; while two releases at a population frequency of 41% result in transgene elimination. C: Population suppression following six consecutive releases of 10,000 males carrying the *Medusa* system (no fitness cost) into a population with a carrying capacity of 10,000 and a growth rate of 9.1. Results depicted are a single run of the stochastic simulation. As the population becomes increasingly transgenic, the number of females declines, resulting in a population crash within ∼12 generations. D: *Medusa* release thresholds as a function of fitness cost under the: a) deterministic model, b) stochastic model where a fitness cost corresponds to an increase in mosquito mortality rate, and c) where it corresponds to a decrease in female mosquito fertility rate. For the deterministic model, release thresholds correspond to two consecutive releases of transgenic males representing a given population frequency; whereas, for the stochastic model, release thresholds are measured by the number of releases of 10,000 transgenic males (i.e. a population frequency of ∼50%) required to induce a population crash. For the stochastic model, the threshold number of releases was taken as the mode of 11 simulations.

Fig. 2A reveals that, under the deterministic population frequency model, for two consecutive releases in which released males (with no fitness cost) represent 50% of the total population post-release for each of the two releases, the population becomes almost entirely transgenic within eight generations. The population also becomes almost entirely male, which would lead to a population crash in an equivalent stochastic model with discrete population sizes. Fig. 2B demonstrates that, in the absence of a fitness cost, two consecutive releases, each representing 42% of the population, results in gene drive, while two releases each representing 41% of the population results in the system being eliminated. Further simulations reveal a threshold frequency for this release scenario of 41.7%, which increases to 44.0% if the system confers a 5% fitness cost on transgenic males and females, and 46.4% for a 10% fitness cost (Fig. 2D). In any case, under a deterministic, discrete-generation model, two releases at a population frequency of 50% seem adequate to achieve gene drive for realistic fitness costs (up to 17%).

To model a population crash, we implement the stochastic, discrete-time model described in the Methods. Here, the mosquito life cycle is divided into four stages – egg, larval, pupal and adult – with density-dependent mortality occurring at the larval stage. Female adults mate once upon emergence, while adult males can mate throughout their lifespan. The environmental carrying capacity is 10,000 adults and a basic reproductive number of 9.1 [21] means that, at low population densities, a single female mosquito will produce on average 9.1 female offspring that survive to adulthood. The time step of this model is one day, with a generation corresponding to ∼27 days, allowing generations to overlap. In Fig. 2C we see that, for six consecutive releases of 10,000 X*^A^*Y*^B^* males (with no fitness cost) spaced half a generation apart, the proportion of transgenic individuals gradually increases, eventually exceeding the threshold required for gene drive. At this point, the transgene is driven into the population and the number of females declines accordingly. This results in a population crash within ∼12 generations of the last release. Release requirements are elevated under the latter stochastic model because, following an initial release, most adult females have already mated with wild-type males and transgenic males are diluted by emerging juvenile wild-types; however the dynamics are otherwise analogous. Simulations suggest that releases every half generation lead to the smallest absolute release requirements (six half-generational releases of 10,000 X*^A^*Y*^B^* males achieve a population crash c.f. eight generational releases and seven quarter-generational releases of the same quantity). Since both the maternal and zygotic toxins of the *Medusa* system act on the embryonic life stage, fitness effects are most likely to manifest as a reduction in the fecundity of transgenic females. However, both a reduction in transgenic female fecundity and an increase in transgenic adult mortality can be overcome by a small number of additional releases – one additional release of 10,000 X*^A^*Y*^B^* males for a 20% reduction in female fecundity and three additional releases for a 20% increase in adult mortality (Fig. 2D).

### Dynamics in two partially-isolated populations

**Figure 3:**
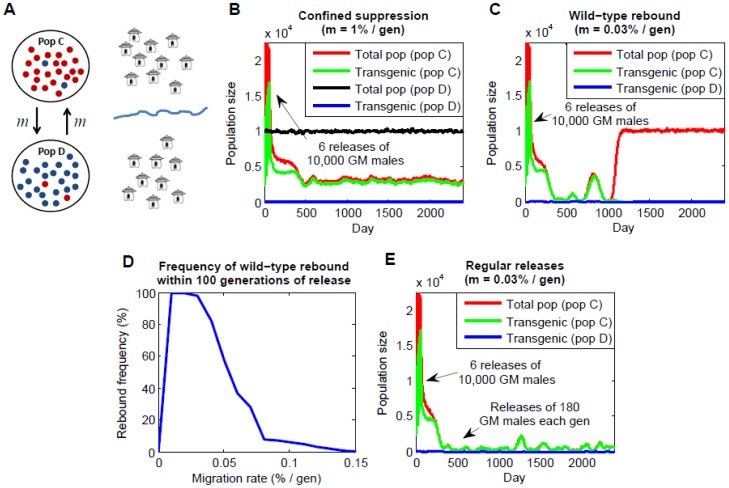
*Medusa* dynamics in two partially-isolated populations. A: Bi-directional migration model in which mosquitoes carrying the *Medusa* system are introduced into population C, and populations C and D exchange a fraction, *m*, of their individuals at each generation. B: Confined population suppression following six consecutive releases of 10,000 *Medusa* males (no fitness cost) into population C (bi-directional migration rate of 1% per generation). Results depicted are a single run of the stochastic simulation. C: Confined population suppression and a wild-type rebound for the same release scenario as in panel B, but a smaller bi-directional migration rate of 0.03% per generation. D: The frequency of a wild-type rebound occurring within 100 generations of a super-threshold release (no *Medusa* fitness cost) as a function of migration rate. E: Sustained population suppression achieved through regular releases of 180 *Medusa* males into population C at each generation following six initial releases of 10,000 *Medusa* males into the same population.

Fig. 2 demonstrates how *Medusa* can be used to induce a population crash in an isolated population; however populations are rarely truly isolated – individuals are usually exchanged between neighboring populations. In this context, two important phenomena must be considered – transgenic contamination of populations neighboring the release population, and flooding of the release population with wild-types as its size declines. We use the bi-directional migration model described in the Methods (Fig. 3A) to explore these issues, assuming no fitness cost as a default scenario. To begin, we consider a bi-directional migration rate of 1% of the source population migrating per generation to reflect migration rates of the malaria vector *An. gambiae* between neighboring villages in Mali as measured by genetic and empirical methods [32]. For a super-threshold release in population C (six consecutive releases of 10,000 transgenic males), the model has two interesting predictions. First, transgenic contamination of population D is minimal (∼0.2%) since *Medusa* never exceeds the threshold for spread in this population. And second, population C never actually crashes but instead undergoes sustained suppression (Fig. 3B). This is because the influx of wild-types from population D prevents population C from crashing while gene drive maintains the *Medusa* system at super-threshold levels. Consequently, the number of females at the release site is persistently reduced by ∼80%.

The sustained suppression seen in Fig. 3B is encouraging; however these dynamics are sensitive to the number of incoming migrants from the wild-type population. The scenario of a reduced migration rate of 0.03% per generation is shown in Fig. 3C. Here, while the *Medusa* system is generally maintained at high frequencies in population C, the population size falls to much lower levels due to the small number of incoming wild-type mosquitoes. This means that, by chance, the number of transgenic mosquitoes can fall to very low levels, eventually falling below the threshold for spread. When this happens, transgenic mosquitoes are quickly driven out of the population, the population ceases to be suppressed, wild-types recolonize and the population returns to its original equilibrium. Due to the stochastic nature of the dynamics, the duration of suppression can vary greatly; but for these parameters, it generally falls in the range of 11–52 generations, with a mean of 28 generations. During the period of suppression, the number of females at the release site is reduced by ∼97% and transgenic contamination of population D is less than 0.01%.

The solution to a rebounding population is a periodic release, the size of which depends on the fitness cost of the *Medusa* constructs, the incoming wild-type migration rate and the frequency of the periodic release. The fact that a migration rate of 1% per generation can prevent a population rebound suggests that regular releases of wild-type males and females are sufficient to prevent a rebound. Fig. 3D depicts the probability of a wild-type rebound within the first 100 generations following a super-threshold release as a function of migration rate. It suggests that a wild-type rebound will almost always occur for a migration rate of 0.03% per generation, it will occur about half the time for a migration rate of 0.05% per generation, and a migration rate of 0.1% per generation will almost always prevent a rebound. However, unfortunately for this strategy, releases of wild-type female mosquitoes are required, and these could potentially transmit vector-borne diseases to humans. This is because the role of the supplementary mosquitoes is to prevent a population crash while maintaining the *Medusa* system above threshold levels, and wild-type females are able to produce transgenic offspring by mating with transgenic males, while wild-type males are only able to produce wild-type offspring (Fig. 1), making them less suitable for this task. This problem is averted through regular releases of transgenic males instead of wild-type males and females. Fig. 3E depicts a scenario whereby an initial super-threshold release of transgenic males is supplemented by a release of 180 additional transgenic males each generation. The number of females at the release site is consequently reduced by ∼98% and transgenic contamination of population D is less than 0.01%. Less frequent releases are possible; but unreliable due to the stochastic nature of small populations and the fact that, once *Medusa* falls below threshold levels, the population is capable of rebounding very quickly, requiring much larger releases to maintain suppression. Even for bi-generational releases, the releases size must be increased five-fold. Generational releases of X*^A^*Y*^B^* males are therefore an efficient means of maintaining population suppression with *Medusa* in the face of wild-type immigrants.

### Comparison to sterile insect technique and autosomal X-shredders

One of the disadvantages of self-limiting systems such as genetically sterile males is that they require frequent and large releases in order to maintain suppression; however, it is likely that suppression with *Medusa* will also require frequent releases to protect against the possibility of a population rebound. A comparison of *Medusa* to these systems is therefore justified. Here, we briefly investigate the dynamics of two such systems: i) female-specific RIDL (Release of Insects carrying a Dominant Lethal) [31]; and ii) X-shredders located on an autosome [20].

**Figure 4:**
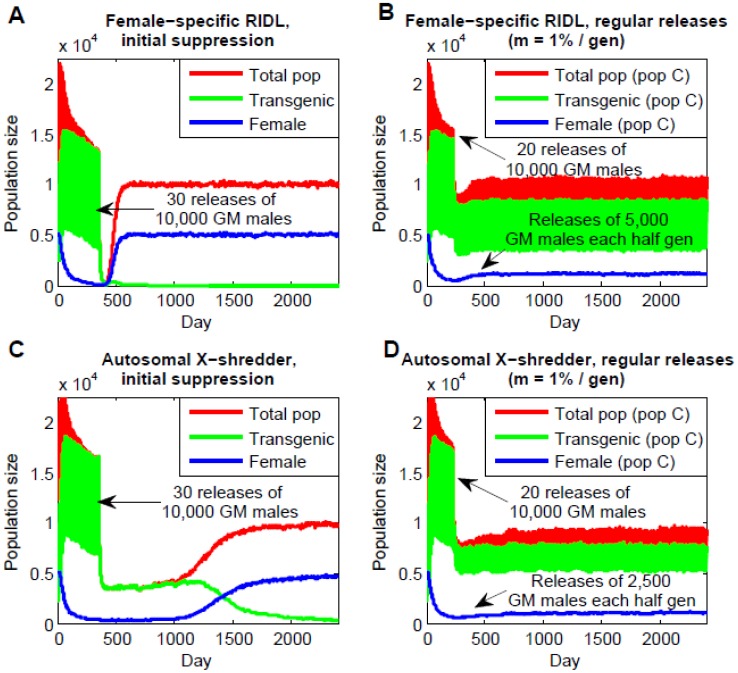
Population dynamics of female-specific RIDL and autosomal X-shredders. A: Initial population suppression following 30 consecutive releases of males homozygous for the female-specific RIDL allele (no fitness cost) into an isolated population. Results depicted are a single run of the stochastic simulation. B: Sustained population suppression achieved through regular releases of 5,000 males homozygous for the female-specific RIDL allele into population C at each half generation following 20 initial releases of 10,000 transgenic males (bi-directional migration rate of 1% per generation). C: Initial population suppression following 30 consecutive releases of males homozygous for the autosomal X-shredder allele (no fitness cost, transgenic males have 90% male offspring) into an isolated population. D: Sustained population suppression achieved through regular releases of 2,500 males homozygous for the autosomal X-shredder allele into population C at each generation following 20 initial releases of 10,000 transgenic males (bi-directional migration rate of 1% per generation).

Female-specific RIDL is a variant of the sterile insect technique in which transgenic female offspring of transgenic males are flightless while transgenic male offspring can fly, thus suppressing the female population while allowing the population-suppressing transgene to persist in the male population for a few generations [31], [33]. We simulated the dynamics of female-specific RIDL in a two-population model analogous to the one we used for the *Medusa* system, assuming no fitness cost for purposes of comparison. This revealed that it is very difficult to crash a population under these conditions (carrying capacity of 10,000, basic reproductive number of ∼9.1, overlapping generations); however 20 consecutive releases of 10,000 transgenic males every half generation were sufficient to reduce the female population size by ∼96%, and 30 consecutive releases were capable of reducing the total population to below 10 mosquitoes. That said, if the population is not completely eliminated and releases are discontinued, the transgene is quickly eliminated due to its inherent fitness cost and the population rebounds. This scenario is shown in Fig. 4A for the case of 30 consecutive releases in an isolated population. Here, the population rebounds within five generations of the final release. Similar results are seen for an analogous two-population model with a migration rate of 1% per generation. A variety of models have investigated the increase in release size required for sterile insect programs when wild-type females immigrate into the control area [34]–[36]. In this case, regular releases of ∼5,000 transgenic males per half generation are required to sustain suppression of the female population by ∼80% (c.f. none for *Medusa*). The transgene disperses into population D; but reaches a maximum frequency of less than 1% (Fig. 4B). For a migration rate of 0.05% per generation, regular releases of ∼5,000 transgenic males per half generation are capable of significantly suppressing the population (c.f. releases of 40 transgenic males per half generation for *Medusa*) meaning that, in this case, *Medusa* release requirements are ∼100 times less than those for female-specific RIDL. Transgenic contamination is low in both cases.

Autosomal X-shredders are a self-limiting variant of the Y-linked X-shredders intended for wide-scale population suppression [20]. As for the Y-linked system, the homing endonuclease gene creates a bias towards Y-bearing spermatozoa and hence male offspring; however, since the construct is autosomal, it no longer favors its own inheritance and in fact confers an effective fitness disadvantage due to the reduction in female offspring. The system is therefore self-limiting and a useful test for the components of Y-linked X-shredders. We simulated the dynamics of autosomal X-shredders analogously to those of *Medusa* and female-specific RIDL, assuming no fitness cost and a 90% bias towards Y-bearing spermatozoa among the gametes of transgenic males. As for female-specific RIDL, a population crash is very difficult to achieve; however 20 consecutive releases of 10,000 transgenic males every half generation are sufficient to reduce the female population size by ∼90%, and 30 consecutive releases are capable of reducing it by ∼93%. In an isolated population, suppression can be sustained for a longer period than for female-specific RIDL in the absence of continued releases with a rebound time of ∼32 generations following 30 consecutive releases (Fig. 4C). For a two-population model with a migration rate of 1% per generation, the transgene is eliminated more quickly and reaches a maximum frequency of ∼8% in population D; which is higher than for either *Medusa* or female-specific RIDL, but still relatively low. Regular releases of ∼2,500 transgenic males per half generation are capable of suppressing the female population by ∼80%, which is half the release size required for similar suppression with female-specific RIDL, and releases of 5,000 transgenic males per half generation reduce the female population size by ∼88% (Fig. 4D). For a migration rate of 0.05% per generation, regular releases of ∼2,000 transgenic males per half generation are capable of significantly suppressing the population, meaning that *Medusa* release requirements are ∼50 times less than those for autosomal X-shredders in this case, and are the lowest of all three confinable systems in all scenarios examined.

## Discussion

We describe a novel gene drive system, *Medusa*, for confined suppression of insect populations in which males are the heterogametic sex, with emphasis on the malaria vector *An. gambiae*. As a gene drive system, *Medusa* could provide a proof of principle for invasive population suppression systems. It could also provide a useful addition to the current repertoire of self-limiting genetic suppression systems such as sterile insect technique, RIDL and autosomal X-shredders. Its benefits over these systems are that it is self-perpetuating and has much smaller release requirements, both in terms of the initial release sizes required to suppress the population, and the subsequent releases required to maintain suppression. Even in the presence of significant fitness costs, nine 1∶1 releases of *Medusa* males to wild mosquitoes should be sufficient to suppress the population, and if subsequent releases are required, they are only a fraction of those required for other self-limiting population suppression systems. Care should be taken before emphasizing differences in release requirements too much since the difference in cost between rearing 50 or 5,000 mosquitoes every half generation may be less than expected once additional costs involved in developing and testing GM mosquito strains, establishing mass rearing facilities and entomological surveillance are accounted for [12]. That said, one of the primary benefits of the *Medusa* system is that, in the presence of sufficient numbers of incoming wild-type individuals, subsequent releases may not be required at all.

Population suppression with *Medusa* is well suited to *An. gambiae*, the main African malaria vector, for two reasons – first, *An. gambiae* has well-studied X and Y chromosomes on which the constructs could be inserted; and second, it disperses quickly over the range of a single village [32], [37], reducing the chance of small-scale population structure stochastically eliminating the transgene and catalyzing a population rebound. Population suppression in *Ae. aegypti* would be more challenging since maleness is thought to be determined by a dominant allele at a single locus, M [38], which is yet to be identified. The Y-linked cassette would need to be tightly linked to this locus, perhaps through creation of an inversion, as this would eliminate the possibility for recombination between the two loci. The X-linked cassette would also need to be located at an equivalent position on the homologous chromosome lacking the M allele, for the same reason. Furthermore, *Ae. aegypti* disperses over much smaller distances, leading to a high degree of population structure even within a single community [39]. This creates increased opportunities for *Medusa* to fall below threshold levels in a partially-isolated sub-population, resulting in a population rebound. While *Medusa* applies to species for which males are heterogametic, an analogous gene drive system, *Merea*, is predicted to induce confined suppression in species for which females are heterogametic (ZW) and males are homogametic (ZZ) [40], if located on the Z chromosome of such species.

It should be noted that the effects of population structure have not been addressed in this modelling study, and more-detailed models will be required to assess the performance of *Medusa* in specific ecological settings. Previous modelling studies have addressed the effects of population aggregation in *Ae. aegypti* on genetic control programs using RIDL [41]–[44] and these have found aggregation to significantly increase release requirements for these programs. However, at least for *An. gambiae*, mark-release-recapture experiments suggest mosquito populations to be well-mixed on the village scale [32], [37], partially justifying the treatment of neighboring villages as panmictic units. That said; *An. gambiae* is a species complex consisting of several chromosomal forms with limited gene flow between them and unique seasonal adaptations, leading to additional spatiotemporal structure considerations [32], [45], [46]. These must be considered if future releases are planned. The larval density-dependence model used in this study is very simplistic, assuming a monotonic increase in larval mortality with density. Data for *Ae. aegypti* suggests a nonlinear relationship in which density-dependence may be over-compensating [47], which has been shown to influence the predicted outcome of genetic control programs. For instance, for sterile male releases or RIDL constructs that kill prior to larval competition, the adult mosquito density is predicted to increase under some scenarios [41], [43], which is potentially problematic for toxin-antidote systems such as *Medusa* that cause zygotic toxicity. Limited data is available for *An. gambiae*; however a recent study by Muriu *et al.* [48] under semi-natural conditions suggests a monotonic decrease in pupation probability with larval density. We have opted for a monotonic density-dependence model; however the dependence of model predictions on factors such as these highlights the importance of modeling real interventions on a species and location-specific basis. Furthermore, it is crucial that ecological experiments better characterize phenomena such as density-dependence.

Finally, a promising aspect of the *Medusa* system is that many of its constituent components are already available and have been shown to work, albeit only in Drosophila. Synthetic *Medea* selfish genetic elements consist of a maternal toxin and a tightly linked zygotic antidote. These have been engineered in *Drosophila* through maternal expression of miRNAs designed to silence maternal expression of a gene whose product is required for normal embryonic development (the toxin), coupled with zygotic expression of a transgene capable of providing the missing activity to those embryos that inherit the construct [28], [49]. Recent work of ours has shown that toxin-antidote pairs of this type can function well together even when their component genes are located on different chromosomes. The UD^MEL^ system, for instance, consists of two sets of maternal toxins with corresponding zygotic antidotes on opposite chromosomes and has been shown to display threshold properties in laboratory gene drive experiments [50].

In other work, we have demonstrated that zygotic toxin-zygotic antidote pairs can be created and function well together in *Drosophila*. For example, expression of the cell death activators Hid (head involution defective), Reaper or Grim under the control of an eye-specific promoter results in blind flies – a trait which can be suppressed through the expression of engineered miRNAs located on an independent construct at another position in the genome [51]. Other zygotic toxin-zygotic antidote pairs can be imagined involving the expression of miRNA toxins designed to silence the expression of an essential endogenous gene and miRNA-insensitive proteins that restore this activity. Additionally, site-specific nuclease-mediated gene integration technologies now make it possible to insert genes at any desired genomic position in a species whose genome has been sequenced and for which transgenesis is possible [52]. Thus, it should be possible to generate X and Y-linked insertions for *Medusa* constructs in many insect pest and disease vector species with X-Y sex determination systems.

Y chromosomes are often largely heterochromatic, which presents challenges to creating Y-linked transgene cassettes with robust and consistent gene expression. That said, a number of genes located in constitutive heterochromatin are expressed at high levels, indicating this is possible [53]. In addition, a gene on the *An. gambiae* Y chromosome expressed in the early embryo (a time window relevant for antidote expression) has also recently been identified [54]. Finally, sequence characteristics common to genes located in constitutive heterochromatin, such as reduced C-G content, are being identified [55]–[57], as are sequences able to bring about boundary formation between heterochromatin and euchromatin [58], [59]. While tests of sufficiency remain to be carried out for the ability of any of these sequences to permit robust transcription in constitutive heterochromatin, it seems reasonable to hope some combination of elements will allow for regulated expression from the Y chromosome.

**Figure 5:**
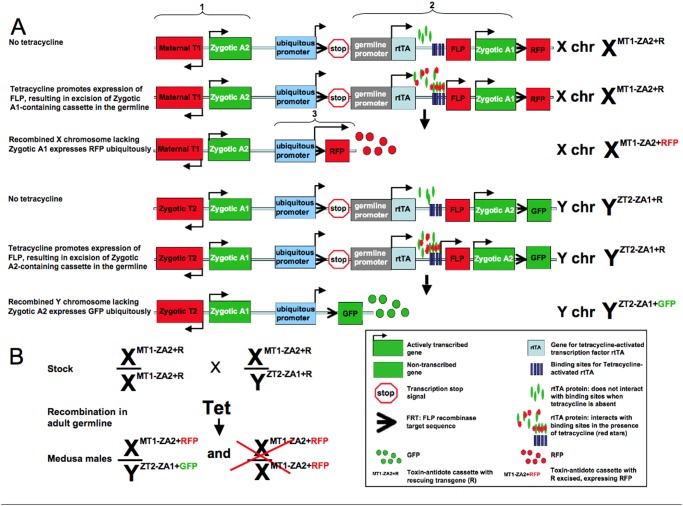
Generating *Medusa* males. A: To generate flies carrying the *Medusa* X chromosome (top), a construct carrying two cassettes (regions enclosed by brackets), each consisting of multiple genes, is introduced onto the X chromosome. Cassette 1 consists of maternal toxin 1 (Maternal T1) and zygotic antidote 2 (Zygotic A2). Cassette 2 carries zygotic antidote 1 (Zygotic A1). It also carries a set of flanking genes that can be used to excise Zygotic A1 from the chromosome in response to the presence of tetracycline. This is achieved as follows: a germline-restricted promoter drives the expression of the rtTA gene, which encodes a tetracycline-dependent transcriptional activator [60]. In the absence of tetracycline (upper chromosome), rtTA does not bind its target sites. Both sexes survive, as do all progeny of females carrying this construct. In the presence of tetracycline (middle chromosome), rtTA binds its target sites and drives the transcription of the FLP recombinase. FLP binds to two target sites (FRTs) and promotes the recombinational excision of the intervening genetic elements, which include a transcriptional stop sequence that prevents a ubiquitous promoter from driving expression of rtTA in all cells, and the rtTA, FLP and Zygotic A1 genes. Excision results in the creation of a chromosome (bottom chromosome) that carries cassette 1 and a newly created cassette 3, which consists of a ubiquitous promoter driving the expression of RFP, a visible marker. Female flies carrying this construct alone are sterile because no progeny inherit zygotic antidote 1, which is needed in order for progeny of mothers carrying maternal toxin 1 to survive. To generate flies carrying the *Medusa* Y chromosome (lower three chromosomes in A), a construct that carries two cassettes, each consisting of multiple genes, is introduced onto the Y chromosome. Cassette 1 consists of zygotic toxin 2 (Zygotic T2) and zygotic antidote 1 (Zygotic A1). Insects carrying this chromosome survive because they also carry cassette 2, which includes zygotic antidote 2 (Zygotic A2). Cassette 2 also carries a set of flanking genes that can be used to excise Zygotic A2 from the Y chromosome in response to the presence of tetracycline. This is achieved as above, for the X chromosome cassette. However, note that males carrying only the excised version of the *Medusa* Y chromosome, and no *Medusa* X chromosome, are dead because they express zygotic T2 but not Zygotic A2. In order to create insects that carry both the excised X cassette and the excised Y cassette, tetracycline-driven excision needs to be done in a context of a cross. B: A stock carrying the non-excised *Medusa* X and Y chromosomes (MT1–ZA2+R and ZT2–ZA1+R, respectively) is viable and fertile (upper, parental generation). When larvae (before differentiation of gametes) carrying these chromosomes are exposed to tetracycline, FLP-mediated recombination occurs in the germline, generating gametes that carry the excised versions of the *Medusa* X and Y chromosomes. Male progeny that inherit excised X and Y chromosomes survive because they carry zygotic antidote 2 as well as zygotic toxin 2. They are identified by the fact that they express both RFP and GFP. Female progeny with excised X chromosomes die because the action of maternal toxin 1 in the adult female is unopposed in progeny by zygotic antidote 1. Progeny that inherit non-excised chromosomes will lack GFP or RFP. Large numbers of *Medusa* males can be sorted away from these using fluorescence-based larval sorting technologies. These are the males used for release.

Provided the above issues related to Y-linked gene expression can be solved, *Medusa*-bearing strains can be generated through the procedure outlined in Fig. 5. In brief, strains are first generated that carry either the X or Y-linked transgenes. In each case, the core *Medusa* gene cassette is linked to a rescue cassette that carries an antidote to the relevant toxin, thereby allowing individuals carrying only one *Medusa* chromosome to be viable (illustrated for X and Y-linked transgenes in Fig. 5A). Importantly, the rescuing antidote is embedded within sequences that allow for conditional excision using FLP recombinase in response to an environmental cue such as exposure to tetracycline or a temperature shift (illustrated for tetracycline exposure in Fig. 5) [61], [62]. These two strains are crossed to each other to establish a stable stock carrying both X and Y-linked *Medusa* elements and associated rescue cassettes (Fig. 5B). When *Medusa* males are desired, adults of this strain are exposed to tetracycline. The only viable progeny, if excision of the rescue constructs has occurred in the parental germline, are *Medusa* males. Progeny males carrying excised versions of both X and Y chromosomes can be identified definitively in large numbers by virtue of the fact that they ubiquitously express two chromosome-specific marker genes that express fluorescent proteins such as GFP or RFP. High throughput sorting of larvae, based on the presence of these markers, can be used to generate large numbers of *Medusa* males for release [63]. It is conceivable that a small number of *Medusa* females may be included in a release intended to be all male; however, simulations suggest that this would make no appreciable difference, the only concern being that these females may be capable of transmitting vector-borne diseases to humans.

As with any form of population control, the presence of *Medusa* will select for resistance; however this can be delayed through multimerization of toxin-encoding genes and insulation of transgenes from the effects of surrounding chromatin [64]. In addition, second-generation *Medusa* elements can be generated that utilize toxin-antidote combinations distinct from those of the first-generation elements [49], making it possible to carry out multiple cycles of population suppression if first-generation elements fail.

Two genetic events could interfere with the spread of *Medusa*. Both of these involve females inheriting the antidote present on the Y-linked construct, thereby preventing the gender-biasing effects of an X-linked maternal toxin and a Y-linked zygotic antidote. First, recombination between X and Y chromosomes could result in the Y-linked antidote moving onto the maternal toxin-bearing X chromosome, enabling the latter to give rise to females capable of producing viable female progeny. That said; this is very unlikely if the transgenes are located in regions of the X and Y chromosomes that normally do not recombine; it would require a chromosome translocation, which is likely to have other associated fitness costs that would prevent it from spreading to high frequency. Second, X chromosomes can become attached to a common centromere, generating a chromosome known as an attached-X which moves as a single unit during meiosis [65]. In *Drosophila*, individuals carrying an attached-X chromosome from their mother and a Y from their father are fertile females (XXY). Female progeny of females bearing the maternal toxin on an attached-X chromosome would be capable of surviving if they inherit the antidote-bearing Y chromosome from their father. The appearance of an attached-X chromosome is also able to prevent sex ratio distortion and population extinction in *Drosophila* carrying a Y-linked segregation distorter [66]. Interestingly, each of these scenarios has the same molecular solution – incorporation of a female-specific toxin onto the transgene-bearing Y chromosome. Such a gene would be silent and invisible to selection when present in males; but when present in females, it would cause death, preventing an attached-X chromosome from interfering with *Medusa* spread.

In conclusion, *Medusa* is an attractive gene drive system for confined suppression of insect populations in which males are the heterogametic sex (in particular, *An. gambiae*, the primary African malaria vector). *Medusa* could provide an important test for the concept of gene drive-mediated population suppression and could serve as an efficient system for local, sustainable and reversible population control. Further modeling will be required to identify insects whose population structure and ecology is best suited for this drive mechanism. The components required to build *Medusa* have been shown to function in *D. melanogaster*, and similar components could presumably be engineered in related species such as the invasive fruit crop pest *Drosophila suzukii* [67], [68], and in less closely-related species, such as mosquitoes and other disease vectors.

## Methods

### Modeling Medusa population dynamics

To characterize the basic dynamics of the *Medusa* system, we consider the element as a single allele on the X chromosome, which we denote by X*^A^*, and a single allele on the Y chromosome, which we denote by Y*^B^*. We refer to the corresponding positions on the wild-type chromosomes as X*^a^* and Y*^b^*, respectively. We then use two modeling frameworks – a deterministic, discrete-generation population frequency framework and a stochastic, discrete-time framework – to model the spread of the element through a population, assuming random mating and 100% toxin efficiency. The assumption of 100% toxin efficiency is justified by the ability to zygotically express long double-stranded RNA or miRNAs that silence the expression of genes essential for early embryo development, leading to unviable offspring [69], and to create synthetic *Medea* elements that show 100% maternal-effect lethality [28], [49].

### Deterministic, discrete-generation population frequency framework

If releases always consist of males having both constructs (i.e. releases are always X*^A^*Y*^B^*), then the dynamics are significantly simplified because X*^A^*Y*^b^* and X*^a^*Y*^B^* males are never generated (Fig. 1). Similarly, if females are never released (the most likely scenario since female mosquitoes transmit diseases to humans and female pest insects lay eggs that cause damage) then X*^A^*X*^A^* females are never generated. This means that the only genotypes we need to consider are X*^A^*Y*^B^*, X*^a^*Y*^b^*, X*^A^*X*^a^* and X*^a^*X*^a^*. We denote the proportions of the 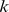 th generation that are males of genotypes X*^A^*Y*^B^* and X*^a^*Y*^b^* by 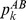, 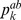, and the proportions that are females of genotypes X*^A^*X*^a^* and X*^a^*X*^a^* by 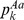 and 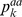, respectively. By considering all possible mating pairs (Fig. 1), the genotype frequencies in the next generation are then given by
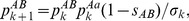
(1)




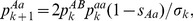
(2)




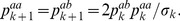
(3)


Here, 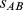 and 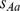 represent the fitness costs associated with X*^A^*Y*^B^* males and X*^A^*X*^a^* females, respectively, and 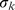 is a normalizing term representing the proportion of embryos that survive to maturity. This is given by,
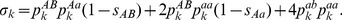
(4)


We refer to the release frequency as the proportion of the total population that are released individuals post-release. For an initial release of *Medusa* males at a population frequency of *x*, we have the initial condition, 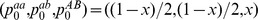, and for a subsequent release at the same population frequency, we make the substitution 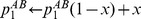 and multiply all other genotype frequencies by 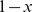.

### Stochastic, discrete-time framework

We can study the population frequency dynamics to get an idea of the threshold properties and basic time-series dynamics of the *Medusa* system; however, to explore the idea of using *Medusa* to induce a population crash, a stochastic model is more appropriate because it can handle a zero population size and the chance events that occur in small populations. Density-dependence is also an important consideration because, at low population sizes, larval competition is reduced and a single female can produce more offspring that survive to adulthood.

Using *An. gambiae* as a case study, we adapt the modeling framework of Deredec *et al.* [21], which itself is based on the population dynamic framework of Hancock and Godfray [70], to describe the spread of the *Medusa* system through a discrete, density-dependent population in discrete time with time steps of one day. In this model, the mosquito life cycle is divided into four life stages – egg, larva, pupa and adult (both male and female) – denoted by the subscripts *E*, *L*, *P* and *M*, respectively. The daily, density-independent mortality rates for the juvenile stages are assumed to be identical and are given by 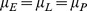, while the duration of these stages differ and are given by 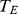, 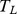 and 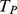. The probability of surviving any of the juvenile stages in a density-independent setting is given by 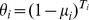, where 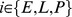; however additional density-dependent mortality, 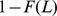, occurs at the larval stage, reducing the probability of surviving this stage by a factor, 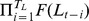. Here, we use a density-dependent equation of the form, 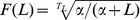, where 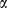 is a parameter influencing the strength of density-dependence. For adult mosquitoes, mortality rates are allowed to differ according to genotype, and are denoted by 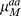, 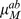, 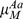 and 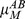. Fecundity rates are also allowed to differ, with wild-type females laying 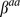 eggs and transgenic females laying 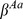 eggs per day. Parameter values are provided in Table S1 in Text S1.

With this framework in place, the dynamics of the population can be described by equations for the number of larvae and adults having each genotype at time *t*. The number of larvae is needed to determine the strength of density-dependence, and for genotype X*^A^*Y*^B^* at time *t*, is given by,
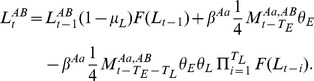
(5)


Here, the first term accounts for survival of X*^A^*Y*^B^* larvae (denoted at time *t* by 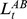) from one day to the next, the second term accounts for newly hatching X*^A^*Y*^B^* eggs from X*^A^*X*^a^* females that have mated with X*^A^*Y*^B^* males (denoted at time *t* by 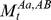, a quarter of the embryos from which have genotype X*^A^*Y*^B^*), and the third term accounts for transformation of larvae into pupae for juvenile stages resulting from the same cross. Equations for the three other genotypes are treated analogously and are shown in Text S1.

Adult males and females are treated slightly differently in this framework since it is assumed that female mosquitoes only mate once, while male mosquitoes may mate throughout their lifetime. For example, the number of male adults of genotype X*^A^*Y*^B^* at time *t* is given by,
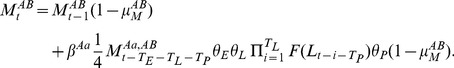
(6)


Here, the first term accounts for survival of X*^A^*Y*^B^* adults (denoted at time *t* by 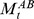) from one day to the next, and the second term accounts for transformation of X*^A^*Y*^B^* pupae into adults, where these pupae result from crosses between X*^A^*X*^a^* females and X*^A^*Y*^B^* males. Females, on the other hand, are assumed to mate only once and on the same day that they emerge. They can therefore be described by both their genotype and the genotype of the male with whom they mated. For example, the number of female adults at time *t* of genotype X*^A^*X*^a^* that have mated with X*^A^*Y*^B^* males is given by,
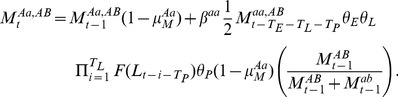
(7)


Here, the first term accounts for survival of X*^A^*X*^a^* adult females that have mated with X*^A^*Y*^B^* males (denoted at time *t* by 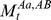) from one day to the next, and the second term accounts for transformation of X*^A^*X*^a^* pupae into adults, where these pupae result from crosses between X*^a^*X*^a^* females and X*^A^*Y*^B^* males (half of the embryos from which have genotype X*^A^*Y*^a^*). This term is multiplied by the fraction of the adult male population having the genotype X*^A^*Y*^B^*. Equations for all other adult genotypes are treated analogously and are shown in Text S1.

These equations can be modified to accommodate the random effects at low population sizes when *Medusa* is causing significant population suppression. We assume that the number of eggs produced per day by each mated genotype follows a Poisson distribution with a mean equal to the fecundity of the female genotype multiplied by the number of females having that mated genotype. The number of eggs having each genotype then follows a multinomial distribution with probabilities given by the inheritance pattern shown in Fig. 1. All survival/death events follow a Bernoulli distribution where the probability of survival is 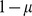 and is specific to each developmental stage and genotype. When applied at the population level, these events follow a Binomial distribution. Finally, female mate choice follows a binomial distribution with probabilities given by the relative frequencies of the two male genotypes in the population.

To model confinement of the *Medusa* system to an isolated population and immigration of wild-type individuals into the release population, we consider a metapopulation model consisting of two populations – population C and population D – each of which exchanges a fraction, *m*, of its adult population with the other at each generation [30]. Genotype numbers in each population are given the subscripts C and D. Transgenic males are released into population C, while population D initially consists of wild-types. The two-population dynamics are then simply accommodated by selecting adults of each genotype to migrate from population C to D according to a Poisson distribution with a mean equal to their population size in population C multiplied by the migration rate *m*, and repeating the same calculation for adults migrating from population D to C. For an equilibrium adult mosquito density of *M_eq_* in both populations and an initial release of *M_eq_ Medusa* males into population C, we have the initial condition,
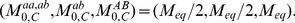
(8)




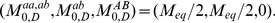
(9)where subscripts C and D represent the corresponding populations. A subsequent release of *X Medusa* males in population C at time *s* is modeled by making the substitution, 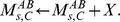


Finally, to compare the dynamics of *Medusa* to female-specific RIDL and autosomal X-shredders, we adapt the inheritance pattern and dynamics described above to accommodate their systems. For female-specific RIDL, we follow the modeling framework of Alphey *et al*. [71]. For autosomal X-shredders, we follow the framework of Deredec *et al.* [21] and consider an autosomal inheritance pattern in which transgenic males produce mostly Y-bearing spermatozoa and hence mostly male offspring. For comparative purposes, we consider the case of no fitness cost. Matlab code implementing these simulations is provided in File S1.

## Supporting Information

File S1 Matlab code implementing simulations for the *Medusa* system.
(M) Click here for additional data file.

Text S1 Model equations and parameter values for the stochastic, discrete-time frameworks for *Medusa*, female-specific RIDL and autosomal X-shredders.
(PDF)
