# Supplementary material for: Medusa: A Novel Gene Drive System for Confined Suppression of Insect Populations
Source: PLoS One. 2014 Jul 23;9(7):e102694. doi: 10.1371/journal.pone.0102694 (PMC4108329; doi:10.1371/journal.pone.0102694)
Supplement: Text S1 — Model equations and parameter values for the stochastic, discrete-time frameworks for Medusa, female-specific RIDL and autosomal X-shredders. (PDF) [file pone.0102694.s002.pdf]

Text S1:

Model equations for *Medusa* stochastic, discrete-time framework:

In the main text we described a stochastic framework for modeling the spread of *Medusa* through a randomly mating population; however we left out several equations for brevity. These are included here for completeness.

Equation 5 in the main text describes the number of larvae of genotype X^AY^B over time. Analogous equations for the other larval genotypes are provided here:

$$L_t^{Aa} = L_{t-1}^{Aa}(1-\mu_L)F(L_{t-1}) + \beta^{aa}\frac{1}{2}M_{t-T_E}^{aa,AB}\theta_E - \beta^{aa}\frac{1}{2}M_{t-T_E-T_L}^{aa,AB}\theta_E\theta_L\prod_{i=1}^{T_L}F(L_{t-i}) \ , \qquad \text{(S1)}$$

$$L_t^{ab} = L_{t-1}^{ab}(1-\mu_L)F(L_{t-1}) + \beta^{aa}\frac{1}{2}M_{t-T_E}^{aa,ab}\theta_E - \beta^{aa}\frac{1}{2}M_{t-T_E-T_L}^{aa,ab}\theta_E\theta_L\prod_{i=1}^{T_L}F(L_{t-i}) \ , \qquad \text{(S2)}$$

$$L_t^{aa} = L_{t-1}^{aa}(1-\mu_L)F(L_{t-1}) + \beta^{aa}\frac{1}{2}M_{t-T_E}^{aa,ab}\theta_E - \beta^{aa}\frac{1}{2}M_{t-T_E-T_L}^{aa,ab}\theta_E\theta_L\prod_{i=1}^{T_L}F(L_{t-i}) \ . \qquad \text{(S3)}$$

In each case, the first term accounts for survival of larvae having the given genotype from one day to the next, the second term accounts for newly hatching eggs of this genotype, and the third term accounts for transformation of larvae of the same genotype into pupae. All symbols are defined in Table S1 and the Methods section of the manuscript. X^AX^a eggs are produced by X^aX^a females that have mated with X^AY^B males (half of the embryos from which have genotype X^AX^a), while X^aX^a females that have mated with X^aY^b males produce eggs that are half X^aX^a and half X^aY^b.

Equation 6 in the main text describes the number of adult males of genotype X^AY^B over time. The other adult male genotype, X^aY^b, is described by the following equation:

$$M_t^{ab} = M_{t-1}^{ab}(1-\mu_M^{ab}) + \beta^{aa}\frac{1}{2}M_{t-T_E-T_L-T_P}^{aa,ab}\theta_E\theta_L\prod_{i=1}^{T_L}F(L_{t-i-T_P})\theta_P(1-\mu_M^{ab}) \ . \qquad \text{(S4)}$$

Here, the first term accounts for survival of X^aY^b adults (denoted at time *t* by M_t^{ab}) from one day to the next, and the second term accounts for transformation of X^aY^b pupae into adults, where these pupae result from crosses between X^aX^a females and X^aY^b males. As described in the main text, females are assumed to mate only once and on the same day that they emerge so can therefore be described by both their genotype and the genotype of the male with whom they mated. Equation 7 describes the number of female adults of genotype X^AX^a that have mated with X^AY^B males over time, and the other mated female genotypes are described by the following equations:

$$M_t^{Aa,ab} = M_{t-1}^{Aa,ab}(1-\mu_M^{Aa}) + \beta^{aa}\frac{1}{2}M_{t-T_E-T_L-T_P}^{aa,AB}\theta_E\theta_L\prod_{i=1}^{T_L}F(L_{t-i-T_P})\theta_P(1-\mu_M^{Aa})\left(\frac{M_{t-1}^{ab}}{M_{t-1}^{AB}+M_{t-1}^{ab}}\right), \tag{S5}$$

$$M_t^{aa,AB} = M_{t-1}^{aa,AB}(1-\mu_M^{aa}) + \beta^{aa}\frac{1}{2}M_{t-T_E-T_L-T_P}^{aa,ab}\theta_E\theta_L\prod_{i=1}^{T_L}F(L_{t-i-T_P})\theta_P(1-\mu_M^{aa})\left(\frac{M_{t-1}^{AB}}{M_{t-1}^{AB}+M_{t-1}^{ab}}\right), \tag{S6}$$

$$M_t^{aa,ab} = M_{t-1}^{aa,ab}(1-\mu_M^{aa}) + \beta^{aa}\frac{1}{2}M_{t-T_E-T_L-T_P}^{aa,ab}\theta_E\theta_L\prod_{i=1}^{T_L}F(L_{t-i-T_P})\theta_P(1-\mu_M^{aa})\left(\frac{M_{t-1}^{ab}}{M_{t-1}^{AB}+M_{t-1}^{ab}}\right). \tag{S7}$$

For each of these equations, the first term accounts for survival of adult females having the given mated genotype from one day to the next, and the second term accounts for transformation of pupae of the given female genotype into adults. The second term is then multiplied by the fraction of the adult male population having either genotype X^AY^B or X^aY^b, depending on the female mated genotype. X^AX^a pupae emerge from eggs produced by adult X^aX^a females that have mated with X^AY^B males (half of the embryos from which have genotype X^AX^a), and X^aX^a pupae emerge from eggs produced by crosses between X^aX^a females and X^aY^b males (half of the embryos from which have the genotype X^aX^a).

Using these equations, we can derive several basic properties of the population, such as the non-zero equilibrium densities of larvae and adults, and the basic reproductive number – i.e. the

average number of female offspring produced by a single female that survive to adulthood at low population densities – in the absence of genetic control. The basic reproductive number can be defined intuitively as the rate of female egg production multiplied by the life expectancy of an adult mosquito multiplied by the proportion of eggs that will survive through all of the juvenile life stages in the absence of density-dependence. This is given by,

$$R_0 = \frac{\beta^{aa}\theta_E\theta_L\theta_P(1-\mu_M^{aa})}{2\mu_M^{aa}} \quad . \tag{S8}$$

The equilibrium population densities can then be calculated by setting the population densities to be equal across generations in Equations S2-S4 and S7. This leads to the following non-zero equilibria:

$$L_{eq} = \alpha(R_0 - 1) \ , \tag{S9}$$

$$M_{eq} = \frac{2}{\beta^{aa}\theta_E}\left(\frac{1-\sqrt[T_L]{\theta_L / R_0}}{1-(\theta_L / R_0)}\right)\alpha(R_0 - 1) \ , \tag{S10}$$

where L_{eq} and M_{eq} represent the total population equilibria (i.e. $L_{eq} = L_{eq}^{ab} + L_{eq}^{aa}$ and $M_{eq} = M_{eq}^{ab} + M_{eq}^{aa,ab}$). These formulations guide the parameter choices, taken from Deredec *et al.* [1], as shown in Table S1:

Table S1: Parameter values for stochastic, discrete-time model

Symbol:	Parameter:	Value:	References:
Primary parameters:			
T_E	Duration of egg stage	1 day	Depinay *et al.* [2]
T_L	Duration of larval stage	14 days	Depinay *et al.* [2]
T_P	Duration of pupal stage	1 day	Depinay *et al.* [2]
$\mu_E = \mu_L = \mu_P$	Mortality rate of juvenile stages	0.168 /day	Molineaux & Gramiccia [3], Depinay *et al.* [2]
$\mu_M^{aa} = \mu_M^{ab}$	Mortality rate of wild-type adult stage	0.123 /day	Molineaux & Gramiccia [3]
β^{aa}	Daily egg production per female	32 /day	Depinay *et al.* [2]
M_{eq}	Equilibrium adult mosquito density	10,000	This paper
Derived parameters:			
g	Average generation time	27.2 days	$T_E + T_L + T_P + 1/\mu_M^{aa}$
θ_E	Survival probability for egg stage	0.831	$(1-\mu_E)^{T_E}$
θ_L	Survival probability for larval stage	0.076	$(1-\mu_L)^{T_L}$
θ_P	Survival probability for pupal stage	0.831	$(1-\mu_P)^{T_P}$
R_0	Generational population grown rate	9.13	Eq. S8
α	Strength of density-dependent component of larval survival	61,300	Eq. S9-S10

Model equations for female-specific RIDL stochastic, discrete-time framework:

The dynamics of female-specific RIDL and autosomal X-shredders are described in the Results section of the main text; however the model formulation was left out for brevity. They are both included here for completeness. For female-specific RIDL, we represent the RIDL construct as a single autosomal allele, *R*, with a corresponding wild-type allele, *r*. We consider the case in which the "lethality" trait is female-specific flightlessness, which allows larval development (and hence larval density-dependent competition) to continue unhindered, while suppressing the adult female population and allowing the transgene to persist for a few generations through the male line. In this case, the larval population can be described by a single variable, L_t, representing the total larval population at time *t*:

$$L_t = L_{t-1}(1-\mu_L)F(L_{t-1}) + \beta(M_{t-T_E}^{f})\theta_E - \beta M_{t-T_E-T_L}^{f}\theta_E\theta_L \prod\nolimits_{i=1}^{T_L} F(L_{t-i}) \ . \qquad \text{(S11)}$$

The first term of this equation accounts for survival of larvae from one day to the next, the second term accounts for newly hatching eggs, and the third term accounts for transformation of larvae into pupae. Here, M_t^f represents the total adult female population size at time *t* and is given by $M_t^f = M_t^{rr,RR} + M_t^{rr,Rr} + M_t^{rr,rr}$, where $M_t^{rr,RR}$, $M_t^{rr,Rr}$ and $M_t^{rr,rr}$ represent the total number of wild-type adult females that have mated with males having genotypes *RR*, *Rr* and *rr*, respectively.

Adult males having genotypes *RR*, *Rr* and *rr* are denoted by the variables M_t^{RR}, M_t^{Rr} and M_t^{rr}, respectively, and are described by the following equations:

$$M_t^{RR} = M_{t-1}^{RR}(1-\mu_M^{RR}) \ , \qquad \text{(S12)}$$

$$M_t^{Rr} = M_{t-1}^{Rr}(1-\mu_M^{Rr}) + \beta\begin{pmatrix}\frac{1}{2}M_{t-T_E-T_L-T_P}^{rr,RR} \\ +\frac{1}{4}M_{t-T_E-T_L-T_P}^{rr,Rr}\end{pmatrix}\theta_E\theta_L\prod\nolimits_{i=1}^{T_L}F(L_{t-i-T_P})\theta_P(1-\mu_M^{Rr}) \ , \qquad \text{(S13)}$$

$$M_t^{rr} = M_{t-1}^{rr}(1-\mu_M^{rr}) + \beta \begin{pmatrix} \frac{1}{2} M_{t-T_E-T_L-T_P}^{rr,rr} \\ +\frac{1}{4} M_{t-T_E-T_L-T_P}^{rr,Rr} \end{pmatrix} \theta_E \theta_L \prod_{i=1}^{T_L} F(L_{t-i-T_P}) \theta_P (1-\mu_M^{rr}) \ . \qquad \text{(S14)}$$

The first term of each of these equations accounts for survival of adult males having the given genotype from one day to the next, and the second term of equations S13 and S14 accounts for transformation of pupae of the given genotype into adults. *RR* males cannot be generated through mating since only wild-type females are viable and hence at least one wild-type allele is always inherited among the next generation. Male *Rr* pupae emerge from eggs produced by wild-type adult females that have mated with *RR* males (half of the embryos from which have the male *Rr* genotype) and from eggs produced by wild-type adult females that have mated with *Rr* males (a quarter of the embryos from which have the male *Rr* genotype). Wild-type male pupae emerge from eggs produced by wild-type adult females that have mated with wild-type males (half of the embryos from which have the wild-type male genotype) and from eggs produced by wild-type adult females that have mated with *Rr* males (a quarter of the embryos from which have the wild-type male genotype).

As described earlier, females are assumed to mate only once and on the same day that they emerge so can therefore be described by both their genotype and the genotype of the male with whom they mated. Since only wild-type females are viable, there are three resulting equations representing females that have mated with males having the genotypes *RR*, *Rr* or *rr*. These are described by the following equations:

$$\begin{aligned} M_t^{rr,RR} &= M_{t-1}^{rr,RR}(1-\mu_M^{rr}) \\ &+\beta \begin{pmatrix} \frac{1}{2} M_{t-T_E-T_L-T_P}^{rr,rr} \\ +\frac{1}{4} M_{t-T_E-T_L-T_P}^{rr,Rr} \end{pmatrix} \theta_E \theta_L \prod_{i=1}^{T_L} F(L_{t-i-T_P}) \theta_P (1-\mu_M^{rr}) \left(\frac{M_{t-1}^{RR}}{M_{t-1}^{RR} + M_{t-1}^{Rr} + M_{t-1}^{rr}} \right) , \end{aligned} \qquad \text{(S15)}$$

$$M_t^{rr,Rr} = M_{t-1}^{rr,Rr}(1-\mu_M^{rr})$$
$$+\beta\begin{pmatrix}\frac{1}{2}M_{t-T_E-T_L-T_P}^{rr,rr} \\ +\frac{1}{4}M_{t-T_E-T_L-T_P}^{rr,Rr}\end{pmatrix}\theta_E\theta_L\prod_{i=1}^{T_L}F(L_{t-i-T_P})\theta_P(1-\mu_M^{rr})\left(\frac{M_{t-1}^{Rr}}{M_{t-1}^{RR}+M_{t-1}^{Rr}+M_{t-1}^{rr}}\right), \qquad \text{(S16)}$$

$$M_t^{rr,rr} = M_{t-1}^{rr,rr}(1-\mu_M^{rr})$$
$$+\beta\begin{pmatrix}\frac{1}{2}M_{t-T_E-T_L-T_P}^{rr,rr} \\ +\frac{1}{4}M_{t-T_E-T_L-T_P}^{rr,Rr}\end{pmatrix}\theta_E\theta_L\prod_{i=1}^{T_L}F(L_{t-i-T_P})\theta_P(1-\mu_M^{rr})\left(\frac{M_{t-1}^{rr}}{M_{t-1}^{RR}+M_{t-1}^{Rr}+M_{t-1}^{rr}}\right). \qquad \text{(S17)}$$

For each of these equations, the first term accounts for survival of adult females having the given mated genotype from one day to the next, and the second term accounts for transformation of wild-type female pupae into adults. The second term is then multiplied by the fraction of the adult male population having either genotype *RR*, *Rr* or *rr*, depending on the female mated genotype. As for males, *Rr* pupae emerge from eggs produced by wild-type adult females that have mated with *RR* males (half of the embryos from which have the female *Rr* genotype) and from eggs produced by wild-type adult females that have mated with *Rr* males (a quarter of the embryos from which have the female *Rr* genotype). Wild-type female pupae emerge from eggs produced by wild-type adult females that have mated with wild-type males (half of the embryos from which have the wild-type female genotype) and from eggs produced by wild-type adult females that have mated with *Rr* males (a quarter of the embryos from which have the wild-type female genotype). Stochastic and migratory considerations are identical to those for the *Medusa* system.

Model equations for autosomal X-shredder stochastic, discrete-time framework:

For autosomal X-shredders, we represent the X-shredding homing endonuclease gene (HEG) as a single autosomal allele, *H*, with a corresponding wild-type allele, *h*. The HEG creates a bias among male gametes towards Y-bearing spermatozoa (we denote the proportion of Y-berating spermatozoa among male gametes by the symbol *c*). This leads to an excess of male offspring, hence decreasing the females population size and the total population size since there are less females to produce eggs. Since this gender distortion is irrelevant at the larval stage, we can use

equation S8 again to describe the total larval population size at time *t*; however, this time, the total adult female population size is given by,

$$M_t^f = M_t^{HH,HH} + M_t^{HH,Hh} + M_t^{HH,hh} + M_t^{Hh,HH} + M_t^{Hh,Hh} + M_t^{Hh,hh} \\ M_t^{hh,HH} + M_t^{hh,Hh} + M_t^{hh,hh} \quad , \tag{S18}$$

where the first genotype in the superscript represents the female genotype, and the second genotype represents the genotype of the male with whom they mated.

Adult males having genotypes *HH*, *Hh* and *hh* are denoted by the variables M_t^{HH}, M_t^{Hh} and M_t^{hh}, respectively, and are described by the following equations:

$$M_t^{HH} = M_{t-1}^{HH}(1-\mu_M^{HH}) + \begin{pmatrix} E_{HH,HH}^{HH,m} + E_{HH,Hh}^{HH,m} \\ +E_{Hh,HH}^{HH,m} + E_{Hh,Hh}^{HH,m} \end{pmatrix} \theta_E \theta_L \prod_{i=1}^{T_L} F(L_{t-i-T_P}) \theta_P (1-\mu_M^{HH}) \ , \tag{S19}$$

$$M_t^{Hh} = M_{t-1}^{Hh}(1-\mu_M^{Hh}) + \begin{pmatrix} E_{HH,hh}^{Hh,m} + E_{HH,Hh}^{Hh,m} + E_{Hh,HH}^{Hh,m} \\ +E_{Hh,Hh}^{Hh,m} + E_{Hh,hh}^{Hh,m} \\ +E_{hh,Hh}^{Hh,m} + E_{hh,HH}^{Hh,m} \end{pmatrix} \theta_E \theta_L \prod_{i=1}^{T_L} F(L_{t-i-T_P}) \theta_P (1-\mu_M^{Hh}) \ , \tag{S20}$$

$$M_t^{hh} = M_{t-1}^{hh}(1-\mu_M^{hh}) + \begin{pmatrix} E_{hh,hh}^{hh,m} + E_{hh,Hh}^{hh,m} \\ +E_{Hh,hh}^{hh,m} + E_{Hh,Hh}^{hh,m} \end{pmatrix} \theta_E \theta_L \prod_{i=1}^{T_L} F(L_{t-i-T_P}) \theta_P (1-\mu_M^{hh}) \ . \tag{S21}$$

The first term of each of these equations accounts for survival of adult males having the given genotype from one day to the next, and the second term accounts for transformation of pupae of the given genotype into adults. Since all crosses are viable – only the ratio of male to female offspring is altered by the HEG – there are up to seven crosses that can generate a given genotype and so we denote the number of male and female eggs of genotype *x* produced by adult females of genotype *y* that have mated with a male of genotype *z* by $E_{y,z}^{x,m}$ and $E_{y,z}^{x,f}$, respectively. These quantities are time-dependent and the product of the fecundity of the female genotype, β^y, the number of females having the given mated genotype, $M^{y,z}$, and the proportion of offspring of this mated genotype having the male or female genotype *z*. If the paternal genotype

is transgenic, then the proportion of male offspring is *c*; however if the father is wild-type, both genders are produced in equal numbers. The numbers of eggs from each cross are given by the following equations:

$$(E_{HH,HH}^{HH,m}, E_{HH,HH}^{HH,f}) = \beta^{HH} M_{t-T_E-T_L-T_P}^{HH,HH} \left(c, 1-c\right) , \tag{S22}$$

$$(E_{HH,Hh}^{HH,m}, E_{HH,Hh}^{HH,f}, E_{HH,Hh}^{Hh,m}, E_{HH,Hh}^{Hh,f}) = \beta^{HH} M_{t-T_E-T_L-T_P}^{HH,Hh} \left(\frac{c}{2}, \frac{1-c}{2}, \frac{c}{2}, \frac{1-c}{2}\right) , \tag{S23}$$

$$(E_{HH,hh}^{HH,m}, E_{HH,hh}^{HH,f}) = \beta^{HH} M_{t-T_E-T_L-T_P}^{HH,hh} \left(\frac{1}{2}, \frac{1}{2}\right) , \tag{S24}$$

$$(E_{Hh,HH}^{HH,m}, E_{Hh,HH}^{HH,f}, E_{Hh,HH}^{Hh,m}, E_{Hh,HH}^{Hh,f}) = \beta^{Hh} M_{t-T_E-T_L-T_P}^{Hh,HH} \left(\frac{c}{2}, \frac{1-c}{2}, \frac{c}{2}, \frac{1-c}{2}\right) , \tag{S25}$$

$$\begin{pmatrix} E_{Hh,Hh}^{HH,m}, E_{Hh,Hh}^{HH,f}, E_{Hh,Hh}^{Hh,m}, \\ E_{Hh,Hh}^{Hh,f}, E_{Hh,Hh}^{hh,m}, E_{Hh,Hh}^{hh,f} \end{pmatrix} = \beta^{Hh} M_{t-T_E-T_L-T_P}^{Hh,Hh} \left(\frac{c}{4}, \frac{1-c}{4}, \frac{c}{2}, \frac{1-c}{2}, \frac{c}{4}, \frac{1-c}{4}\right) , \tag{S26}$$

$$(E_{Hh,hh}^{Hh,m}, E_{Hh,hh}^{Hh,f}, E_{Hh,hh}^{hh,m}, E_{Hh,hh}^{hh,f}) = \beta^{Hh} M_{t-T_E-T_L-T_P}^{Hh,hh} \left(\frac{1}{4}, \frac{1}{4}, \frac{1}{4}, \frac{1}{4}\right) , \tag{S27}$$

$$(E_{hh,HH}^{Hh,m}, E_{hh,HH}^{Hh,f}) = \beta^{hh} M_{t-T_E-T_L-T_P}^{hh,HH} \left(c, 1-c\right) , \tag{S28}$$

$$(E_{hh,Hh}^{Hh,m}, E_{hh,Hh}^{Hh,f}, E_{hh,Hh}^{hh,m}, E_{hh,Hh}^{hh,f}) = \beta^{hh} M_{t-T_E-T_L-T_P}^{hh,Hh} \left(\frac{c}{2}, \frac{1-c}{2}, \frac{c}{2}, \frac{1-c}{2}\right) , \tag{S29}$$

$$(E_{hh,hh}^{hh,m}, E_{hh,hh}^{hh,f}) = \beta^{hh} M_{t-T_E-T_L-T_P}^{hh,hh} \left(\frac{1}{2}, \frac{1}{2}\right) . \tag{S30}$$

Adult females are described according to their mated genotype by the following equations:

$$\begin{aligned} &(M_t^{HH,HH}, M_t^{HH,Hh}, M_t^{HH,hh}) = (M_{t-1}^{HH,HH}, M_{t-1}^{HH,Hh}, M_{t-1}^{HH,hh})(1-\mu_M^{HH}) \\ &+\begin{pmatrix} E_{HH,HH}^{HH,f} + E_{HH,Hh}^{HH,f} + \\ E_{Hh,HH}^{HH,f} + E_{Hh,Hh}^{HH,f} \end{pmatrix} \theta_E \theta_L \prod_{i=1}^{T_L} F(L_{t-i-T_P}) \theta_P (1-\mu_M^{HH}) \frac{(M_{t-1}^{HH}, M_{t-1}^{Hh}, M_{t-1}^{hh})}{M_{t-1}^{HH} + M_{t-1}^{Hh} + M_{t-1}^{hh}} , \end{aligned} \tag{S31}$$

$$\begin{aligned} &(M_t^{Hh,HH}, M_t^{Hh,Hh}, M_t^{Hh,hh}) = (M_{t-1}^{Hh,HH}, M_{t-1}^{Hh,Hh}, M_{t-1}^{Hh,hh})(1-\mu_M^{Hh}) \\ &+\begin{pmatrix} E_{HH,hh}^{Hh,f} + E_{HH,Hh}^{Hh,f} + E_{Hh,HH}^{Hh,f} \\ +E_{Hh,Hh}^{Hh,f} + E_{Hh,hh}^{Hh,f} \\ +E_{hh,Hh}^{Hh,f} + E_{hh,HH}^{Hh,f} \end{pmatrix} \theta_E \theta_L \prod_{i=1}^{T_L} F(L_{t-i-T_P}) \theta_P (1-\mu_M^{Hh}) \frac{(M_{t-1}^{HH}, M_{t-1}^{Hh}, M_{t-1}^{hh})}{M_{t-1}^{HH} + M_{t-1}^{Hh} + M_{t-1}^{hh}} , \end{aligned} \tag{S32}$$

$$(M_t^{hh,HH}, M_t^{hh,Hh}, M_t^{hh,hh}) = (M_{t-1}^{hh,HH}, M_{t-1}^{hh,Hh}, M_{t-1}^{hh,hh})(1-\mu_M^{hh})$$
$$+\begin{pmatrix} E_{hh,hh}^{hh,f} + E_{hh,Hh}^{hh,f} \\ +E_{Hh,hh}^{hh,f} + E_{Hh,Hh}^{hh,f} \end{pmatrix} \theta_E \theta_L \prod_{i=1}^{T_L} F(L_{t-i-T_P}) \theta_P (1-\mu_M^{hh}) \frac{(M_{t-1}^{HH}, M_{t-1}^{Hh}, M_{t-1}^{hh})}{M_{t-1}^{HH} + M_{t-1}^{Hh} + M_{t-1}^{hh}} \quad \text{(S33)}$$

For each of these equations, the first term accounts for survival of adult females having the given mated genotype from one day to the next, and the second term accounts for transformation of pupae of the given female genotype into adults. The second term is then multiplied by the fraction of the adult male population having either genotype *HH*, *Hh* or *hh*, depending on the female mated genotype. Stochastic and migratory considerations are again identical to those for the *Medusa* system.

References:

1. Deredec A, Godfray HCJ, Burt A (2011) Requirements for effective malaria control with homing endonuclease genes. Proc. Natl. Acad. Sci. USA 108: 874-880.
2. Depinay JMO, Mbogo CM, Killeen G, Knols B, Beier J, et al. (2004) A simulation model of African *Anopheles* ecology and population dynamics for the analysis of malaria transmission. Malar. J. 3: 29.
3. Molineaux L, Gramiccia G (1980) The Garki Project: Research on the Epidemiology and Control of Malaria in the Sudan Savanna of West Africa. Geneva: World Health Organization Press. 311 p.
